# Optimal use of biologics with endoscopic balloon dilatation for repeated intestinal strictures in Crohn's disease

**DOI:** 10.1002/jgh3.12329

**Published:** 2020-03-28

**Authors:** Akihito Uda, Hiroyo Kuwabara, Sayuri Shimizu, Ryuichi Iwakiri, Kiyohide Fushimi

**Affiliations:** ^1^ Department of Health Policy and Informatics, Graduate School of Medical and Dental Sciences Tokyo Medical and Dental University Tokyo Japan; ^2^ Healthcare Management Research Center Chiba University Hospital Chiba Japan; ^3^ Research Department Institute for Health Economics and Policy Tokyo Japan; ^4^ Department of Medicine Saga University Saga Japan

**Keywords:** antitumor necrosis factor alpha, Crohn's disease, immunomodulator, intestinal strictures, observational study, steroid

## Abstract

**Background and Aim:**

Intestinal strictures in Crohn's disease (CD) have a high rate of repeated surgery. As alternatives to surgery, endoscopic balloon dilatation (EBD), immunomodulators (IMs), and antitumor necrosis factor alpha (anti‐TNFα) have been proposed. We aimed to assess the effectiveness of the combined therapy with anti‐TNFα and EBD in preventing intestinal stricture recurrence and surgery in patients with CD.

**Methods:**

This retrospective cohort study included patients from the nationwide administrative database in Japan who were hospitalized and underwent at least one EBD between 1 April 2010 and 31 March 2017. The effectiveness of anti‐TNFα was evaluated by performing survival analysis for the primary outcome. We selected the inverse probability of treatment weighting method for adjustment of covariates. As an exploratory analysis, we evaluated the association of anti‐TNFα initiation timing with intestinal stricture recurrence.

**Results:**

The anti‐TNFα exposed group had a significantly lower risk of intestinal stricture recurrence than that of the anti‐TNFα nonexposed group (hazard ratio = 0.38, 95% confidence interval 0.31–0.48, *P* < 0.001). Surgery‐free rate was shown to have the same tendency. Anti‐TNFα therapy initiation before or after EBD resulted in a lower risk of intestinal stricture recurrence than that of simultaneous treatment.

**Conclusion:**

The combined therapy with anti‐TNFα and EBD could have preventive effects for intestinal stricture recurrence and surgery in hospitalized patients with CD. In particular, anti‐TNFα initiation may be recommended before or after EBD, not immediately after EBD. With respect to EBD, it is important to clarify the effectiveness of combination therapy with several new medication treatments, such as biologics.

## Graphical abstract

The combined therapy with anti‐TNFα and EBD could have preventive effects for intestinal stricture recurrence and surgery in hospitalized patients with CD. Particularly, anti‐TNFα initiation may be recommended before or after EBD, not immediately after EBD. With respect to EBD, it is important to clarify the effectiveness of combination therapy with several new medication treatments, such as biologics.

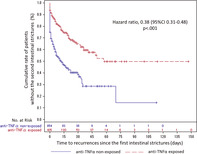

## Introduction

Enteropathy, often associated with bleeding, strictures, and occasionally perforations, has gained increased clinical attention in the recent years because of the advancement of endoscopic technologies, such as capsule and single‐/double‐balloon endoscopy, which enable easier detection of mucosal lesions in the small intestine.^1^, ^2^ Intestinal strictures are a frequent complication in patients with Crohn's disease (CD) and are of great clinical importance and require adequate treatment. From the viewpoint of the natural history of CD, the incidence of strictures tends to gradually increase since disease onset (approximately 10–15% within 5 years and 15–20% within 20 years).^3^ Their clinical importance is clearly highlighted by the high rate of surgical resections.^4^, ^5^ Patients without improvement in their intestinal obstructive symptoms with medication treatment are recommended to undergo surgery. Approximately 80% of patients with CD will eventually require at least one surgical resection within 10 years from diagnosis.^5^, ^6^ Surgery is remarkably effective in improving the obstructive symptoms; however, endoscopic CD recurrence may occur within 1 year postsurgery, requiring additional surgery in at least 34% of cases.^5^, ^7^, ^8^ In addition, surgical intervention might increase the recurrence rate of intestinal strictures.^9^

Several therapeutic options have been proposed as an alternative to surgery, such as endoscopic balloon dilatation (EBD) and therapy with immunomodulators (IMs) and antitumor necrosis factor alpha (anti‐TNFα). In a previous meta‐analysis, 58% of patients with CD who underwent EBD were surgery‐free for 33 months, which was the mean follow‐up period, and the immediate success rate of EBD was satisfactory in terms of avoiding urgent surgery.^10^ IMs and anti‐TNFα were established to treat intestinal strictures to avoid repeated surgical resection and the related risk of short bowel syndrome, and to reduce the economic burden on the patients and society.^11^, ^12^, ^13^ In particular, anti‐TNFα could dramatically change the treatment strategy for CD. However, it is yet unclear whether the early use of anti‐TNFα could improve the natural history of CD, even though the annual surgical rates tend to decrease worldwide.^14^, ^15^, ^16^, ^17^ There is also controversy regarding whether anti‐TNFα therapy, particularly infliximab, would result in intestinal stricture progression. According to the TREAT study (The Crohn's Therapy Resource Evaluation and Assessment Tool), which surveyed the long‐term safety of infliximab mainly in North America, infliximab itself was not a risk factor for the occurrence of intestinal strictures.^18^ The ACCENT I trial, a randomized, double‐blind, placebo‐controlled trial of anti‐TNFα chimeric monoclonal antibody (Infliximab, Remicade) in the long‐term treatment of patients with moderately to severely active CD, showed results similar to those of the TREAT study.^19^ However, few cases have shown improvement in fibrous intestinal strictures after medication treatment; unlike edematous strictures, fibrous intestinal strictures require surgical treatment.

Furthermore, due to continuous obstructive symptoms, some patients with CD require repeated EBD or surgery, which—as invasive treatments—affect their quality of life. There is no sufficient evidence showing the benefit of medication treatment, such as anti‐TNFα, in patients suspected of having inflammatory strictures because this syndrome is difficult to define and diagnose; hence, these patients might have been excluded from clinical trials. Thus, it is important to prevent the repeated recurrence of intestinal strictures by developing an optimal combination therapy with medication treatment and endoscopic procedures. In this study, we aimed to assess the association between anti‐TNFα prescription with EBD and the recurrence of intestinal strictures in patients with CD from a pharmacoepidemiologic perspective.

## Methods

### *Study design*

In this retrospective cohort study, we analyzed the data of inpatients with CD registered in the Diagnosis Procedure Combination (DPC) database from 1 April 2010 to 31 March 2017. We explored the use of anti‐TNFα for the prevention of small intestinal stricture recurrence and evaluated the association between intestinal stricture recurrence and anti‐TNFα prescription before recurrence during hospitalization.

### *Study population*

With data collected since 2003, the DPC is a large administrative database that includes anonymized data on inpatient admissions to acute care hospitals in Japan. It contained the data of approximately 1198 hospitals at the end of 2017, which were derived voluntarily from 1667 hospitals, approximately 80% of the total number of beds in the acute hospitals in Japan. The detailed data included in the database are as follows: several diagnostic codes such as main disease name, most charged medical resources and so on, according to the International Statistical Classification of Diseases and Related Health Problems 10th Revision (ICD‐10) coding scheme by the World Health Organization; disease names coded with the Japanese Disease Name Codes; medical procedures coded with the Japanese Procedure Codes; and prescription information containing generic drug names. It should be noted that, when a patient is transferred to another hospital or clinic, this administrative data can no longer be collected continuously.

In this study of the DPC database, we extracted the data for inpatients with diagnosis codes for CD (ICD‐10 codes K50.0, K50.1, K50.8, and K50.9) from 1 April 2010 to 31 March 2017 and at least one EBD of the small intestine and colon during hospitalization within 4 weeks from admission (defined as index date). Before EBD, several medication treatments (steroids, IMs, or anti‐TNFα) and imaging examinations by barium radiography or computed tomography (CT)/magnetic resonance imaging (MRI) are recommended in the clinical guidelines.^20^ Among them, steroids are a traditional choice for inflammation control after admission; their short‐term use is recommended for inflammatory strictures in the clinical guidelines.^20^ Although the duration of steroid use in the induction phase differs in previous studies, the duration for evaluating steroid responsiveness, steroid dependency, or refractivity to some steroids, such as prednisolone, was approximately 4–8 weeks.^21^, ^22^, ^23^, ^24^, ^25^, ^26^ Thus, we only included patients who underwent the first EBD within 4 weeks from admission (index date).

### *Exposure definition*

**Figure 1:**
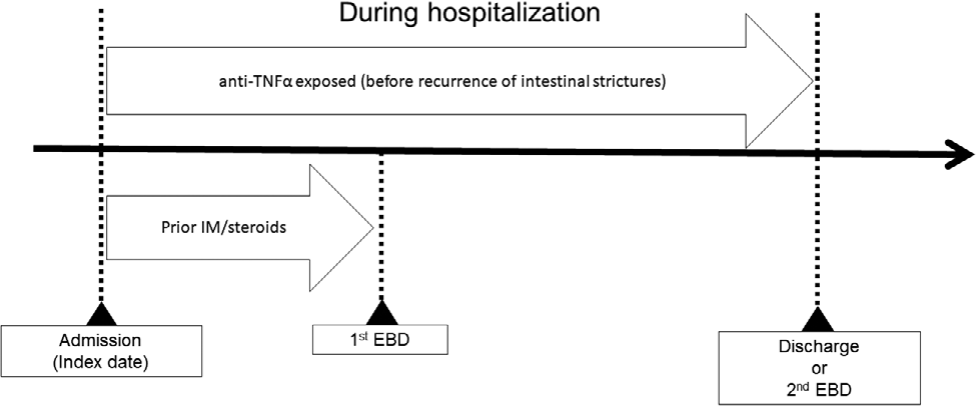
Schematic procedure for this study in selected patients with at least one EBD (*n* = 1289). The index date was defined as the date of admission during hospitalization, and exposure was defined as exposure to anti‐TNFα before the recurrence of intestinal strictures or discharge. Anti‐TNFα, antitumor necrosis factor alpha; EBD, endoscopic balloon dilatation.

For this analysis, we mainly focused on the effectiveness of the combined use of anti‐TNFα (adalimumab or infliximab) and EBD for prevention of intestinal stricture recurrence. The prescription of anti‐TNFα in the period between the index date (admission) and the recurrence of intestinal strictures (second EBD) or discharge was defined as exposure. In addition, combination therapy with anti‐TNFα and EBD from index date to surgery was also defined as exposure for subgroup analysis. Based on the current practice, the initiation timing of anti‐TNFα therapy was defined as before, simultaneously (e.g. 3 days before and after), and after the first EBD. The use of anti‐TNFα after the first EBD was further divided into two periods (within 2 weeks and after >2 weeks from the first EBD). The 2‐week threshold was changed as described in the Statistical Analysis section. The schematic procedure for this study is shown in Figure 1.

### *Outcomes*

The primary outcome measure was defined as the time to intestinal stricture recurrence from the first EBD during the hospitalization. The occurrence of intestinal strictures was defined as EBD for the small intestine and colon. In a subgroup analysis of patients without surgery at index date, we also evaluated the time from index date to surgery in patients with CD who were exposed and not exposed to combined therapy with anti‐TNFα and EBD. For exploratory purposes, the second EBD or surgery after the first EBD was defined as another outcome. In the survival analysis, discharge is usually defined as censored. Discharge was categorized as follows: discharge by disease improvement and discharge by death. We have to consider these two types of discharge because of the competing risk. However, in this study population, there were no deaths. In addition, the mortality rate of patients with inflammatory bowel disease (IBD) is low.^27^, ^28^ Thus, we defined all discharges as censored.

### *Covariates*

Covariates were chosen based on their potential for confounding the results and availability of data in the DPC database. The covariates that were included in the analyses were gender, age, surgery at index date, Charlson comorbidity index, distance from home to hospital, and presence of anal fistula (ICD‐10 code, K60.3) at index date. Furthermore, based on the findings of previous studies, where the use of IMs or steroids prior to EBD was reported to improve the clinical outcomes of intestinal strictures in patients with CD,^24^, ^29^ the prior use of an IM (azathioprine or mercatopurine) or steroids (prednisone, prednisolone, methylprednisolone, budesonide, or betamethasone) was included as a covariate because of their potential effectiveness in preventing intestinal stricture recurrence.

### *Statistical analysis*

The patients' characteristics were presented using standard, descriptive summary statistics, such as means and medians with standard deviation and interquartile range for continuous variables and as proportion for categorical variables.

For the primary outcome, the effectiveness of the combined therapy with anti‐TNFα and EBD was evaluated as the time to intestinal stricture recurrence or discharge using the Kaplan–Meier method. To compare the data between the anti‐TNFα exposed and nonexposed groups, we selected the inverse probability of treatment weighting (IPTW) method with multivariate Cox proportional hazard models to show the adjusted hazard ratios (HRs) by covariates, which were calculated from the inverse of the propensity score using the logistic regression model. In the subgroup of patients without surgery at index date, the time from index date to surgery was also evaluated using the same methods described above.

As an exploratory analysis, we evaluated the association between the anti‐TNFα initiation timing and other factors according to the period of anti‐TNFα initiation, as described in the Exposure definition section, using multivariate Cox proportional hazard models. Furthermore, as a sensitivity analysis, we simulated the influence of the anti‐TNFα initiation timing after the first EBD subsequent to the simultaneous period by changing several different thresholds from 4 to 30 days within a range that could be calculated with sufficient sample size using a spline curve.

*P* value <0.05 was considered statistically significant. All statistical analyses were performed using SAS version 9.4 (SAS Institute, Cary, NC, USA).

This study was approved by the ethics committee of Tokyo Medical and Dental University. Written informed consent was not required because of the anonymous nature of the data.

## Results

### *Patients' characteristics*

During the study period, the study population included 1289 patients (5.6%) from the DPC database who were diagnosed with CD as most charged medical resources and had undergone at least one EBD during the hospitalization among all CD patients having the main diagnosis name based on the definition as described in the Methods section (*n* = 22 962) (Fig. S1).

The baseline patients' characteristics at the index date are shown in Table 1. The mean age was 39.5 and 42.4 years, with male patients accounting for 72.9 and 76.6% of the anti‐TNFα exposed and nonexposed groups, respectively. There were differences among the age category subgroups between the two exposure groups. The proportion of the 18–39 years age subgroup was higher in the anti‐TNFα exposed group (51.7 *vs* 42.3%), and the proportion of the 40–74 years subgroup was higher in the anti‐TNFα nonexposed group (56.0 *vs* 47.4%). In addition, the proportion of surgery at index date was slightly higher in the anti‐TNFα nonexposed group than in the anti‐TNFα exposed group (25.3 *vs* 15.2%, respectively).

**Table 1:** Patients' characteristics at the date of admission (index date), drug exposures prior to the first EBD, and initiation timing of anti‐TNFα in both groups

	Patients with at least one intestinal stricture (*n* = 1289)	
Variable	Anti‐TNFα exposed (*n* = 435)	Anti‐TNFα non‐exposed (*n* = 854)
Baseline characteristics at the index date		
Gender, *n* (%)		
Female	118 (27.1)	200 (23.4)
Male	317 (72.9)	654 (76.6)
Age, years		
Mean (SD)	39.5 (11.4)	42.4 (11.8)
Median (IQR)	39.0 (32.0–46.0)	41.0 (34.0–49.0)
Age category, *n* (%)		
<18	3 (0.7)	2 (0.2)
18–39 years	225 (51.7)	361 (42.3)
40–74 years	206 (47.4)	478 (56.0)
≥75	1 (0.2)	13 (1.5)
Surgery at the index date, *n* (%)		
No	369 (84.8)	638 (74.7)
Yes	66 (15.2)	216 (25.3)
Anal fistula at the index date, *n* (%)		
No	424 (97.5)	842 (98.6)
Yes	11 (2.5)	12 (1.4)
Charlson Comorbidity Index		
Mean (SD)	0.26 (0.6)	0.22 (0.5)
Distance from home to hospital, km		
Mean (SD)	10.4 (9.6)	10.2 (9.8)
Median (IQR)	8.1 (2.8–15.5)	7.6 (2.2–15.2)
Drug exposures prior to the first EBD, *n* (%)		
Immunomodulator, *n* (%)	274 (63.0)	635 (74.4)
Steroids	231 (53.1)	628 (73.5)
Type of anti‐TNFα, *n* (%)		
Adalimumab	202 (46.4)	—
Infliximab	233 (53.6)	—
Initiation timing of anti‐TNFα from the first EBD, *n* (%)		
Prior use	47 (10.8)	—
Simultaneous use	220 (50.6)	—
Within 2 weeks	116 (26.7)	—
More than 2 weeks	52 (12.0)	—

Anti‐TNFα, antitumor necrosis factor alpha; EBD, endoscopic balloon dilatation; IQR, interquartile range.

Drug exposures prior to the first EBD are also shown in Table 1. In the anti‐TNFα nonexposed group, both prior users of IMs and steroids were likely to be prescribed anti‐TNFα (IM: 63.0 *vs* 74.4%; steroids: 53.1 *vs* 73.5%). In the anti‐TNFα exposed group, adalimumab and infliximab showed a similar proportion (46.4 *vs* 53.6%, respectively). Regarding anti‐TNFα initiation timing from the first EBD, simultaneous use had the highest frequency (prior use: 10.8%; simultaneous use: 50.6%; within 2 weeks: 26.7%; >2 weeks: 12.0%).

### *Recurrence rate of intestinal strictures in each exposure group (descriptive analysis)*

Table 2 shows the intestinal stricture recurrence, defined as EBD, as outcomes. The number of patients with recurrent EBD were 102 (23.4%) and 372 (43.6%) in the anti‐TNFα exposed and nonexposed groups, respectively.

**Table 2:** Recurrence of intestinal strictures defined as endoscopic balloon dilatation

	Anti‐TNFα exposed (*n* = 435)	Anti‐TNFα nonexposed (*n* = 854)
Recurrence (−)	333 (76.6)	482 (56.4)
Recurrence (+)	102 (23.4)	372 (43.6)

Anti‐TNFα, antitumor necrosis factor alpha; EBD, endoscopic balloon dilatation.

### *Association between* *anti‐TNFα* *exposure and intestinal stricture recurrence*

**Figure 2:**
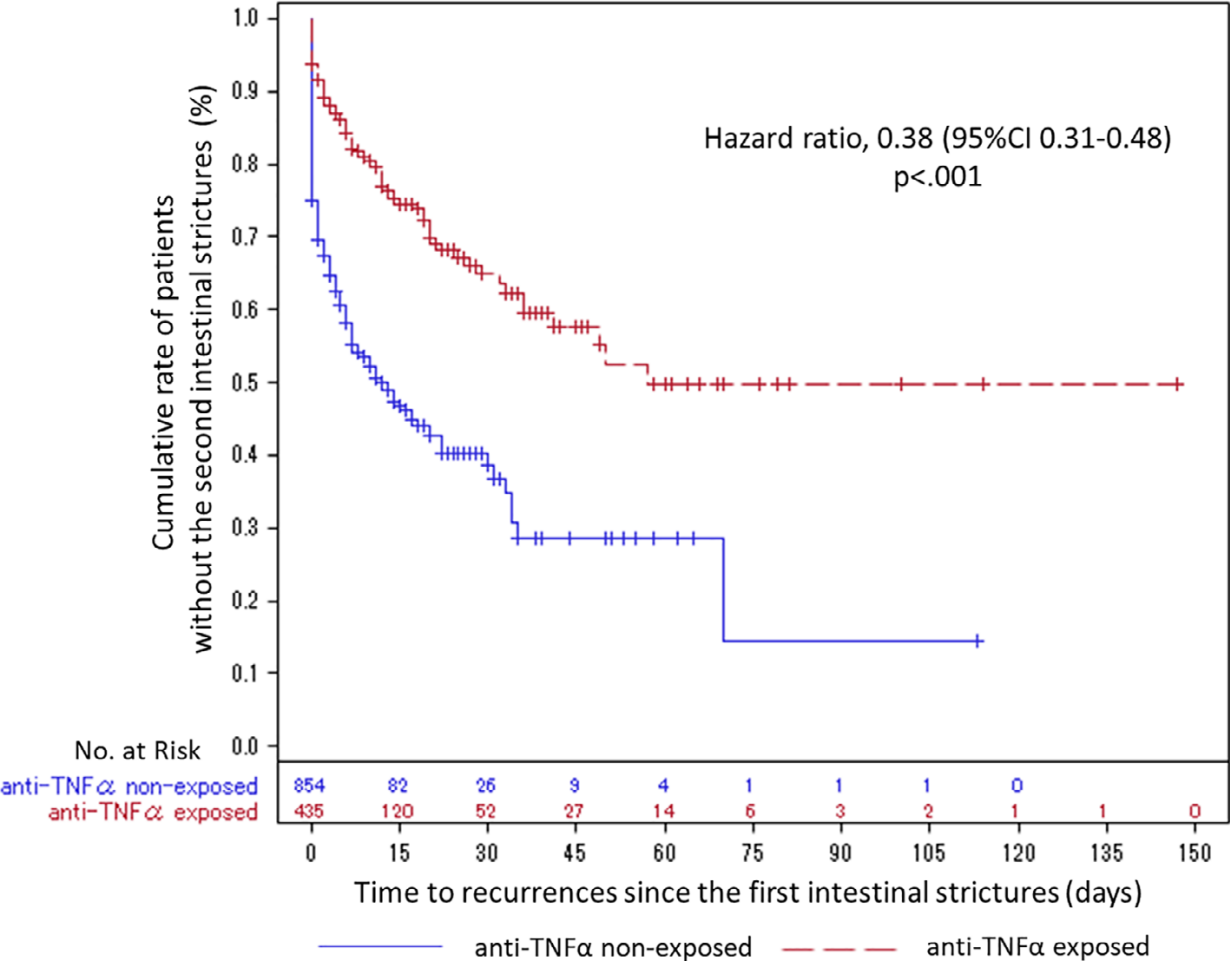
Kaplan–Meier survival curves and HR based on multivariate cox proportional hazard models using inverse probability of treatment weighted (IPTW) for time to recurrence of intestinal strictures from the first intestinal strictures in anti‐TNFα exposed and nonexposed patients (*n* = 1289). Anti‐TNFα, antitumor necrosis factor alpha; CI, confidence interval; HR, hazard ratio.

Figure 2 shows the Kaplan–Meier curve and HR with *P*‐value for the time to intestinal stricture recurrence from the first EBD. The anti‐TNFα therapy tended to have preventive benefits for intestinal stricture recurrence. Based on the multivariate Cox proportional hazard models with IPTW adjustment, the anti‐TNFα exposed group had a significantly lower risk of intestinal stricture recurrence than that of the anti‐TNFα nonexposed group (HR = 0.38, 95% confidence interval [CI] 0.31–0.48, *P* < 0.001). For exploratory purposes, the result from outcomes defined as second EBD or surgery showed the same tendency (HR = 0.39, 95% CI 0.31–0.48, *P* < 0.001) (Fig. S2).

### *Association between combined* *anti‐TNFα/EBD* *therapy and surgery‐free rate (subgroup analysis)*

**Figure 3:**
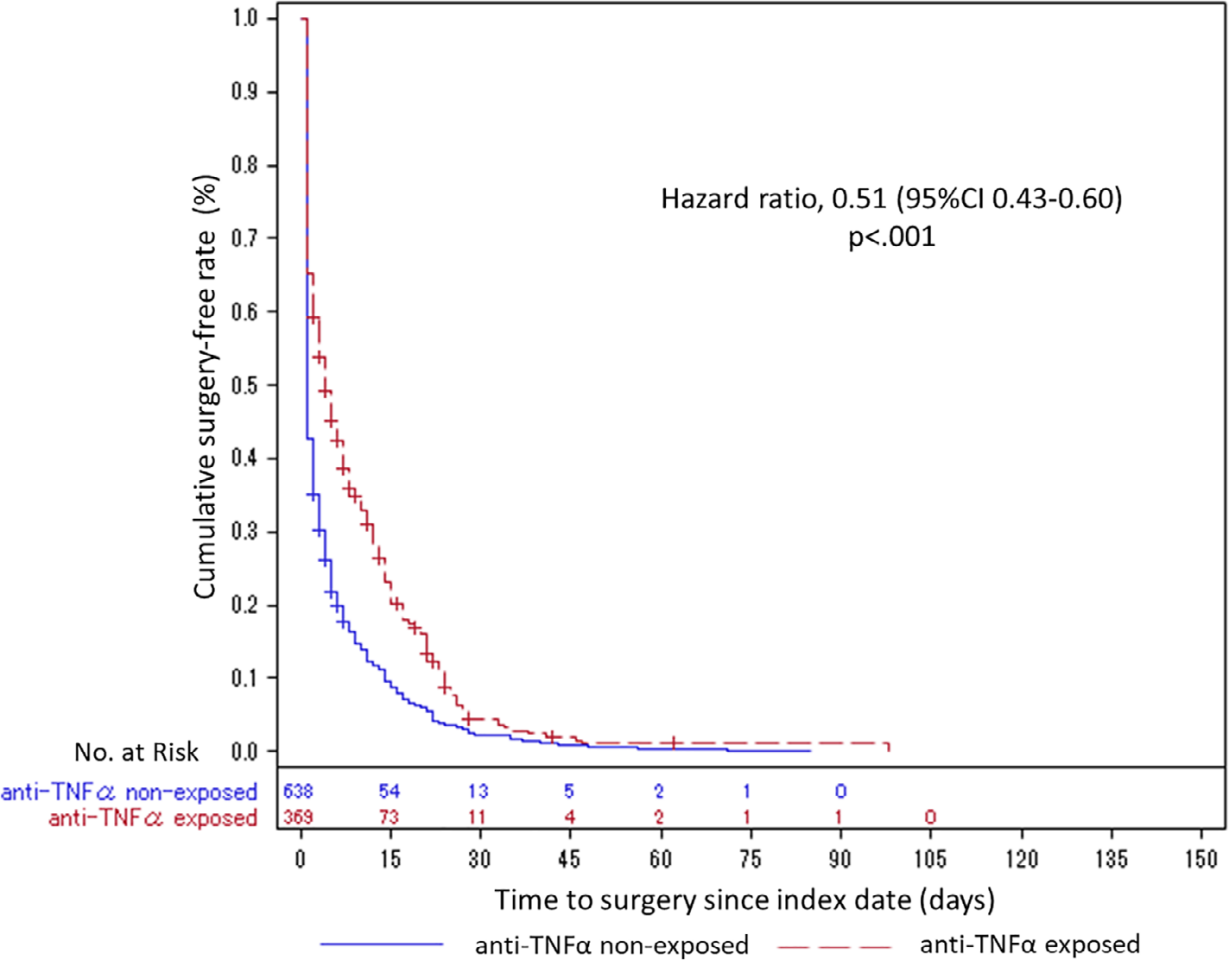
Kaplan–Meier survival curves and HR based on multivariate Cox proportional hazard models using inverse probability of treatment weighted (IPTW) for time to surgery from index date in preoperative anti‐TNFα combined with EBD exposed and nonexposed patients with CD (*n* = 1007). Anti‐TNFα, antitumor necrosis factor alpha; CD, Crohn's disease; CI, confidence interval; EBD, endoscopic balloon dilatation; HR, hazard ratio.

Figure 3 shows the Kaplan–Meier curve and HR with *P*‐value for the time from index date to surgery in the subgroup of patients without surgery at index date. The combined therapy with anti‐TNFα and EBD resulted in longer surgery‐free periods. Based on the multivariate Cox proportional hazard models with IPTW adjustment, patients exposed to combined anti‐TNFα/EBD therapy had a significantly lower risk of surgery than that of the nonexposed group (HR = 0.51, 95% CI 0.43–0.60, *P* < 0.001).

### *Association between the initiation timing of* *anti‐TNFα* *and intestinal strictures recurrence among* *anti‐TNFα* *exposed patients*

**Figure 4:**
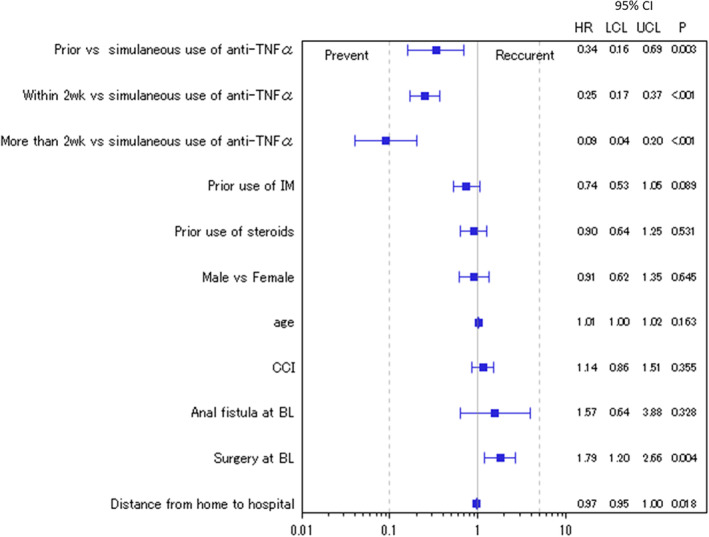
Forest plots for HRs of recurrence of intestinal strictures in each initiation timing of anti‐TNFα and other factors during hospitalization in patients with CD in the anti‐TNFα exposed group (*n* = 435). Anti‐TNFα, antitumor necrosis factor alpha; BL, baseline; CD, Crohn's disease; CCI, Charlson comorbidity index; HR, hazard ratio; IM, immunomodulators; LCL, lower confidence limit; UCL, upper confidence limit.

Patients with anti‐TNFα therapy initiated before or after the first EBD tended to have a lower risk of intestinal stricture recurrence than patients undergoing simultaneous anti‐TNFα/EBD therapy (Fig. 4). The HR was 0.34 (95% CI 0.16–0.69, *P* = 0.003) for patients with anti‐TNFα initiated before the first EBD, 0.25 (95% CI 0.17–0.37, *P* < 0.001) for those with anti‐TNFα initiated within 2 weeks from the first EBD, and 0.09 (95% CI 0.04–0.20, *P* = <0.001) for those with anti‐TNFα initiated after >2 weeks from the first EBD, showing statistical significance. In contrast, the HR was 1.79 (95% CI 1.20–2.66, *P* = 0.004) for patients with surgery at index date.

**Figure 5:**
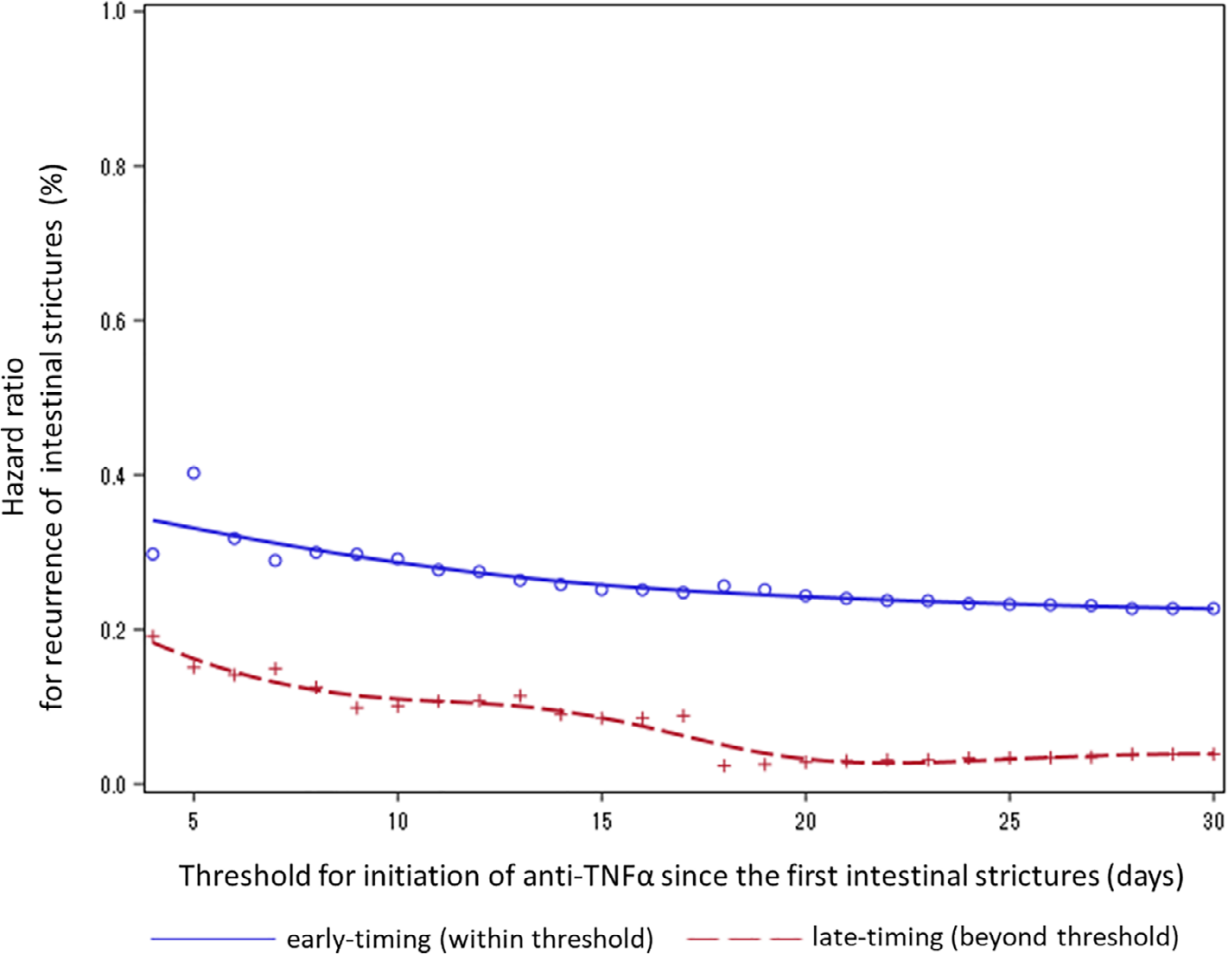
Sensitivity analysis shown as spline curve for hazard ratios of recurrence of intestinal strictures at each timing of anti‐TNFα use after the first EBD to compare “early‐timing” (within the threshold for initiation of anti‐TNFα) with “late‐timing” (beyond the threshold for initiation of anti‐TNFα) during hospitalization in patients with CD who were prescribed anti‐TNFα before the recurrence of intestinal strictures, for the anti‐TNFα exposed group (*n* = 435). Anti‐TNFα, antitumor necrosis factor alpha; CD, Crohn's disease; EBD, endoscopic balloon dilatation.

In the sensitivity analysis, a later anti‐TNFα initiation tended to have greater preventive effects for intestinal stricture recurrence than those of early initiation (Fig. 5).

## Discussion

In our retrospective study of patients with CD who had intestinal strictures during the hospitalization between 2010 and 2017, we demonstrated the preventive effects of the combined therapy with anti‐TNFα and EBD for intestinal stricture recurrence.

The baseline characteristics of the study population were broadly consistent with those in the previous studies for patients with CD in Japan.^30^, ^31^, ^32^ A previous study of CD using the other administrative database (JMDC Inc.) in Japan reported a similar proportion of male patients in the study population.^31^

According to our results, the combined therapy with anti‐TNFα and EBD would have preventive effects on intestinal stricture recurrence in hospitalized patients with severe CD. The surgery‐free rate in these patients was also lower, although it was evaluated in a short‐term period during the hospitalization. In a previous study on anti‐TNFα, ulcerative lesions were markedly improved, but the intestinal stenosis tended to become worse after the patients were administered infliximab.^33^ However, in clinical practice, there have been cases where the properties of the stricture lesions could change through anti‐inflammatory treatment. Therefore, the use of anti‐TNFα may be beneficial to patients undergoing EBD as anti‐TNFα administration decreases edema and shortens the length of strictures.^34^ In addition, another study reported that concomitant EBD during long‐term infliximab therapy had value in improving the clinical outcomes, such as the cumulative surgery‐free rate.^35^ This previous research mainly focused on the efficacy of EBD in patients on maintenance therapy with infliximab who were considered to have controlled or stable disease and were not in a flare‐up phase at the start of the maintenance treatment. Apart from these previous pieces of evidence, we aimed to clarify the clinical requirement for appropriately prescribing anti‐TNFα with concomitant EBD in patients with severe, acute CD during hospitalization. Furthermore, our study has significance as the results were based on a large‐scale database that allowed us to detect rare events, such as intestinal strictures, considering that most previous reports involved small cohorts.

Moreover, careful prescription of anti‐TNFα might be required immediately after EBD for patients with severe, acute CD based on the current results. It is important to consider the initiation time of anti‐TNFα. Small bowel inspection (fluoroscopy or CT/MRI) before anti‐TNFα initiation is useful for distinguishing edematous from fibrous intestinal strictures, facilitating the decision to administer anti‐TNFα. These examinations should be recommended to improve the prognosis (efficacy and safety) through optimal treatment based on accurate diagnosis, although some small bowel inspection modalities, such as CT/MRI, are expensive. In addition to treatment efficacy and safety, we have to consider the medical costs from the viewpoint of health economics. Biologics represent a substantial cost to the health‐care systems, accounting for the majority of CD‐related costs^36^; a previous study has shown increasing costs with the increased use of biologics.^37^ In patients with CD who are at risk of intestinal stricture recurrence, optimal use of biologics, such as anti‐TNFα, will be required after accurate diagnosis with consideration of its efficacy, safety, and economics in both patients and society.

This study has several limitations. First, potential confounding factors were adjusted for in the appropriate multivariate models and the IPTW method; however, controlling all possible variables, including unmeasured confounding variables, was impossible because the data were extracted retrospectively from the administrative database. For example, accurate information on the disease duration, disease symptoms, type of strictures such as mucosal edema (accompanied with inflammation or fibrosis), the size and length of strictures, and disease site (anastomosis site or naïve lesion) were not available, although we only included in‐hospital patients considered to have a severe status. The clinical guidelines recommend that EBD should be considered based on the length and number of the strictures, and the presence of ulcers.^20^ In addition, there were no information on the details of EBD, such as the balloon size, dilatation pressure and time, the ability of passage of stricture site, which might be factors of a long‐term course, and whether EBD was performed for small bowel or colon. Second, we focused on the combination effect of only anti‐TNFα and EBD, not IM, steroids, or nutritional therapy, even though the prior use of IM or steroids, which could be available in the DPC database, was considered as covariate. The efficacy of monotherapy, even with anti‐TNFα, is limited compared to combination therapy. The SONIC (The Study of Biologic and Immunomodulator Naive Patients in Crohn's Disease) and DIAMOND (Deep Remission of Immunomodulator and Adalimumab Combination Therapy for Crohn's Disease) studies investigated the efficacy of combined therapy with anti‐TNFα (infliximab, adalimumab) and IM, respectively, in patients with CD.^38^, ^39^, ^40^, ^41^, ^42^ Further studies are required to evaluate multiple options of combination therapies, including EBD, that would be suitable for individual patients to achieve mucosal healing to prevent intestinal complications and not only to promote remission. Finally, we have defined the outcome as second EBD or surgery. However, some patients had several EBDs during hospitalization. This study could not evaluate that time‐dependent effect for our results. Thus, any results can only be tentative.

In conclusion, the combined therapy with anti‐TNFα and EBD could have preventive effects for intestinal stricture recurrence and surgery in hospitalized patients with CD. In particular, anti‐TNFα initiation may be recommended before or after EBD, not immediately after EBD. With respect to EBD, it is important to clarify the effectiveness of combination therapy with several new medication treatments, such as biologics.

## Declaration of conflict of interest

Akihito Uda, Hiroyo Kuwabara, and Ryuichi Iwakiri were employees of Takeda Pharmaceutical Co. Ltd. when this study was conducted. Sayuri Shimizu and Kiyohide Fushimi have no conflicts of interest to declare.

## Financial support

This study was supported by a Grant‐in‐Aid for Research on Policy Planning and Evaluation from the Ministry of Health, Labour and Welfare, Japan (H30‐Seisaku‐Shitei‐004).

## Supporting information


**Figure S1** Patients' selection for this study (N = 1289). The index date was defined as the date of admission during hospitalization.

**Figure S2** Kaplan–Meier survival curves and HR based on multivariate Cox proportional hazard models using inverse probability of treatment weighted (IPTW) for time to recurrence of intestinal strictures (defined as the second EBD) or surgery from the first intestinal strictures in anti‐TNFα exposed and nonexposed patients (N = 1289). anti‐TNFα, antitumor necrosis factor alpha; HR, hazard ratio; CI, confidence interval, EBD, endoscopic balloon dilatation.
