# Supplementary figures and images for: Optimal use of biologics with endoscopic balloon dilatation for repeated intestinal strictures in Crohn's disease
Source: JGH Open. 2020 Mar 28;4(3):532–40. doi: 10.1002/jgh3.12329 (PMC7273704; doi:10.1002/jgh3.12329)

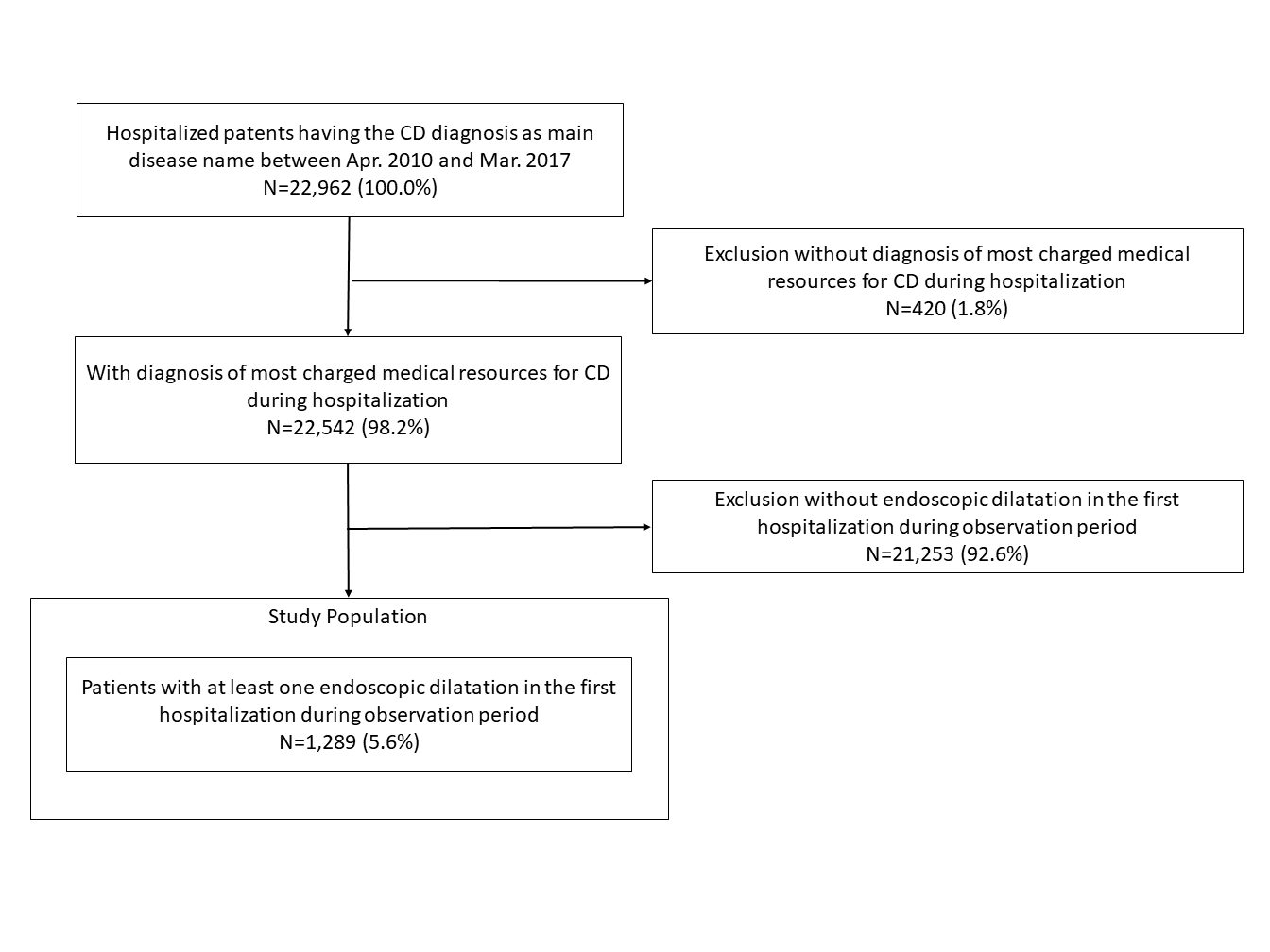

Supplement: Supplementary file 1 — Figure S1 Patients' selection for this study (N = 1289). The index date was defined as the date of admission during hospitalization. [file JGH3-4-532-s001.TIF]

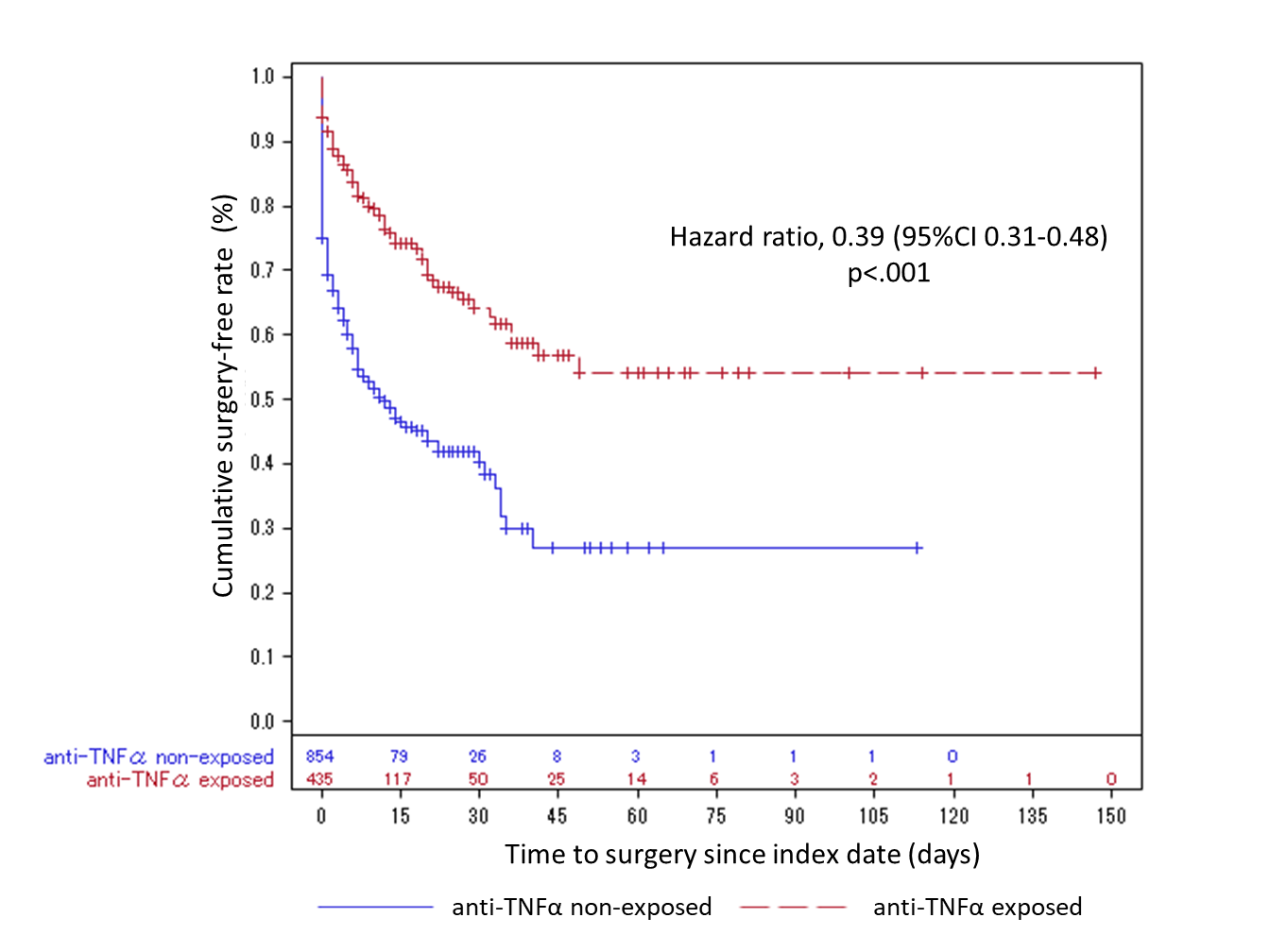

Supplement: Supplementary file 2 — Figure S2 Kaplan–Meier survival curves and HR based on multivariate Cox proportional hazard models using inverse probability of treatment weighted (IPTW) for time to recurrence of intestinal strictures (defined as the second EBD) or surgery from the first intestinal strictures in anti‐TNFα exposed and nonexposed patients (N = 1289). anti‐TNFα, antitumor necrosis factor alpha; HR, hazard ratio; CI, confidence interval, EBD, endoscopic balloon dilatation. [file JGH3-4-532-s002.TIF]
